# Fibroblast Activation Protein Overexpression in Gastrointestinal Tumors: Protocol for a Systematic Review and Meta-analysis

**DOI:** 10.2196/45176

**Published:** 2023-04-26

**Authors:** Yoshiaki Sunami, Ulrich Ronellenfitsch, Jorg Kleeff, Artur Rebelo

**Affiliations:** 1 Department of Visceral, Vascular and Endocrine Surgery University Hospital Halle (Saale) Martin-Luther-University Halle-Wittenberg Halle (Saale) Germany

**Keywords:** fibroblast activation protein, cancer-associated fibroblasts, survival, fibroblast, protein, gastrointestinal, GI, gastrointestinal tumor, cancer, oncology, review method, systematic review, meta-analysis, meta-analyses, cell biology, proteomic

## Abstract

**Background:**

A hallmark of gastrointestinal cancer, especially pancreatic cancer, is the dense stromal tumor microenvironment in which cancer-associated fibroblasts (CAFs) represent the major stromal cell type. Preclinical studies have demonstrated that depletion of fibroblast activation protein (FAP)–positive CAFs results in increased survival.

**Objective:**

We present the protocol for a systematic review and meta-analysis that aim to assess the currently available evidence on the effect of FAP expression on survival and clinical characteristics in gastrointestinal cancers.

**Methods:**

The literature search and data analysis will be conducted in accordance with the PRISMA (Preferred Reporting Items for Systematic Reviews and Meta-Analyses) 2020 statement. The databases PubMed/MEDLINE, Web of Science Core Collection, Cochrane Library, and ClinicalTrials.gov will be searched via their respective online search engines. A meta-analysis comparing patients with and without FAP overexpression with the following outcomes will be performed: postoperative survival (overall and median survival; 1-, 2-, 3-, and 5-year survival rates), histological differentiation (grading), local tumor invasion, lymph node metastases, and distant metastases. Odds ratios will be calculated for binary data, and weighted mean differences and relative SD differences will be determined for continuous data. The 95% CI, heterogeneity measures, and statistical significance will be reported for each outcome. The chi-square and Kruskal-Wallis tests will be used to evaluate statistical significance. A *P* value of <.05 will be considered statistically significant.

**Results:**

Database searches will commence in April 2023. The meta-analysis will be completed by December 2023.

**Conclusions:**

In recent years, several publications on FAP overexpression in gastrointestinal tumors have been published. The only published meta-analysis on this topic dates to 2015. It included 15 studies on various solid tumors and only 8 studies focusing exclusively on gastrointestinal tumors. The expected results of the present analysis will provide new evidence on the prognostic value of FAP in gastrointestinal tumors and thereby support health care professionals and patients in their decision-making.

**Trial Registration:**

PROSPERO CRD42022372194; https://tinyurl.com/352ae8b8

**International Registered Report Identifier (IRRID):**

PRR1-10.2196/45176

## Introduction

Cancers of the gastrointestinal (GI) tract include colorectal, esophageal, liver, pancreas, and stomach cancer. These 5 major types of GI cancer represented 26% of the global cancer incidence and 35% of all cancer-related deaths in 2018 [1]. A hallmark of GI cancers, especially pancreatic cancer, is the dense stromal tumor microenvironment where cancer-associated fibroblasts (CAFs) represent the major stromal cell type [2]. CAFs reprogram the immune microenvironment and promote cancer proliferation, migration, invasion, and metastasis [3,4]. To that end, depleting CAFs has been considered a promising therapeutic strategy for patients with GI cancer. However, in a preclinical pancreatic cancer mouse model, depletion of the major CAF marker α–smooth muscle actin (α-SMA)–positive cells led to invasive, undifferentiated tumors and reduced animal survival [5]. Thus, targeting CAFs may also have tumor-promoting effects due to their functional heterogeneity. Therefore, identification and specific targeting of tumor-promoting CAF subtypes and markers are emerging strategies [6]. CAFs represent highly heterogeneous subpopulations with different functions, which can be both tumor promoting and tumor restraining [7]. To identify and characterize CAF subtypes, a number of markers have been identified, such as desmin, fibroblast activation protein (FAP), fibroblast-specific protein, podoplanin, α-SMA, and vimentin [8,9].

FAP is a type II transmembrane serine protease, which shares a high-sequence identity with dipeptidyl peptidase 4 [10]. In most adult tissues, expression of FAP is low to undetectable. FAP expression becomes highly upregulated in multiple types of cancer, and it is predominantly observed in CAFs [11]. In a preclinical study, it was shown that FAP activates macrophages and promotes liver inflammation and fibrosis [12]. FAP plays a key role in promoting tumor progression and metastasis, further shaping the immunosuppressive tumor microenvironment [13]. Other preclinical studies have demonstrated that pharmacological inhibition or deletion of FAP-positive CAFs results in the attenuation of tumor growth and increased survival in pancreatic cancer models [14,15]. A recent study further consistently showed that tumor-promoting FAP-positive CAFs and tumor-restraining α-SMA–positive CAFs regulate signaling pathways differently [15].

To assess the currently available evidence on the effect of FAP expression on survival and clinical characteristics in GI cancers, we plan to conduct a systematic review and meta-analysis. With this research protocol, we further aim to achieve reproducibility and transparency of our meta-analysis on the published literature as previously demonstrated [16,17].

## Methods

The literature search and data analysis will be conducted in accordance with the PRISMA (Preferred Reporting Items for Systematic Reviews and Meta-Analyses) 2020 statement [18]. The study has been registered in the PROSPERO (International Prospective Register of Systematic Reviews) database (CRD42022372194) [19].

### Search Strategy

**Figure 1:**
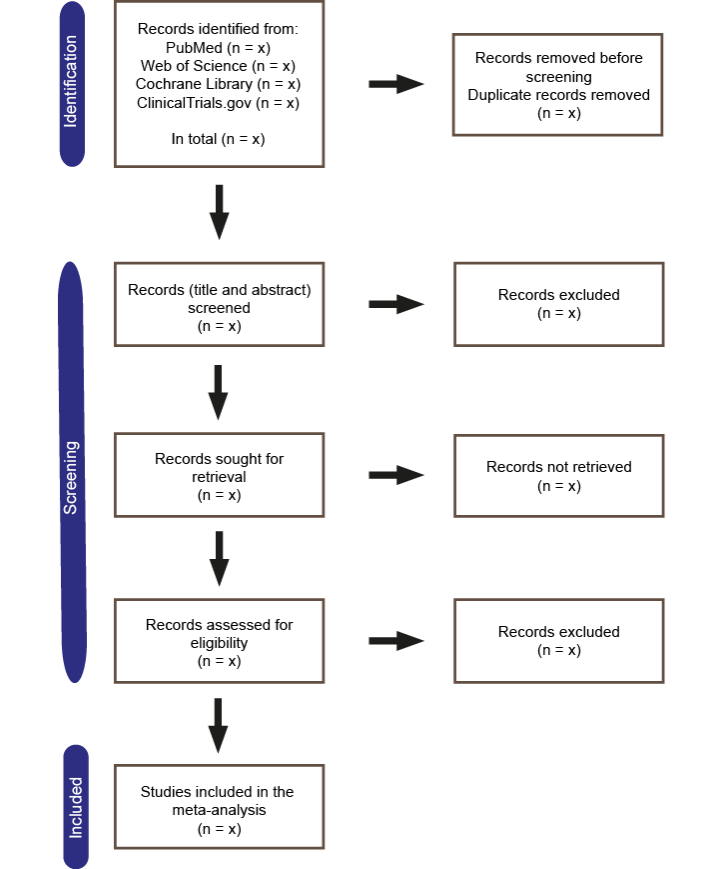
PRISMA (Preferred Reporting Items for Systematic Reviews and Meta-Analyses) flowchart.

The databases PubMed/MEDLINE, Web of Science Core Collection, Cochrane Library, and ClinicalTrials.gov will be searched via their respective online search engines. Citavi 6 (Swiss Academic Software GmbH) will be used as an automatic deduplication system for the studies retrieved from the various databases (Figure 1). The search will be performed on studies published between database inception and a defined search date. The search strategies used in each database are displayed in Table 1.

Titles and abstracts will be evaluated independently in a standardized manner by 2 authors to assess eligibility for inclusion or exclusion. All the potential studies identified from the search will be coded as either “retrieve” (eligible, potentially eligible, or unclear) or “do not retrieve.” For studies coded “retrieve,” 2 reviewers will independently screen the full text and recommend inclusion or exclusion. Disagreements between the reviewers will be resolved by consensus; if no agreement can be reached, a third reviewer will decide whether to include the respective study. The reference lists of the included studies will be manually searched to find additional relevant articles.

**Table 1:** Search strategy.

Database	Search strategy
PubMed/MEDLINE	“surface expressed protease”[tw] OR “seprase”[tw] OR “FAPalpha”[tw] OR “fibroblast activation protein-alpha”[tw] OR “FAP protein”[tw] OR “fibroblast-activating protein”[tw] OR “fibroblast proliferation factor”[tw] OR “fibroblast activation protein, alpha”[tw] OR “fibroblast activation protein”[tw] OR “seprase protein”[tw] AND “neoplasms”[Mesh]
Web of Science Core Collection (all fields)	(“surface expressed protease” OR “seprase” OR “FAPalpha” OR “fibroblast activation protein-alpha” OR “FAP protein” OR “fibroblast-activating protein” OR “fibroblast proliferation factor” OR “fibroblast activation protein, alpha” OR “fibroblast activation protein” OR “seprase protein”) AND (“tumor” OR “neoplasm” OR “tumors” OR “neoplasia” OR “neoplasias” OR “cancer” OR “cancers” OR “malignant neoplasm” OR “malignancy” OR “malignancies” OR “malignant neoplasms” OR “neoplasm, malignant” OR “neoplasms, malignant”)
Cochrane Library (title, abstract, keyword)	(“surface expressed protease” OR “seprase” OR “FAPalpha” OR “fibroblast activation protein-alpha” OR “FAP protein” OR “fibroblast-activating protein” OR “fibroblast proliferation factor” OR “fibroblast activation protein, alpha” OR “fibroblast activation protein” OR “seprase protein”) AND (“tumor” OR “neoplasm” OR “tumors” OR “neoplasia” OR “neoplasias” OR “cancer” OR “cancers” OR “malignant neoplasm” OR “malignancy” OR “malignancies” OR “malignant neoplasms” OR “neoplasm, malignant” OR “neoplasms, malignant”)
ClinicalTrials.gov	*Condition or disease:* Neoplasms *Other terms:* “surface expressed protease” OR “seprase” OR “FAPalpha” OR “fibroblast activation protein-alpha” OR “FAP protein” OR “fibroblast-activating protein” OR “fibroblast proliferation factor” OR “fibroblast activation protein”

### Inclusion and Exclusion Criteria

Only articles in the English language will be considered. Studies comparing patients with and without FAP overexpression in GI tumors and reporting on at least one of the following a priori defined outcomes will be included: postoperative survival (overall and median survival; 1-, 2-, 3-, and 5-year survival rates), histological differentiation (grading), local tumor invasion (as defined in the included studies), lymph node metastases, and distant metastases. Review articles, case reports, case series with less than 5 patients, commentaries, and letters will not be included (Table 2). Details of the study selection process will be summarized in a flowchart according to the recommendations of the PRISMA 2020 statement (Figure 1).

**Table 2:** Inclusion and exclusion criteria.

	Inclusion criteria	Exclusion criteria
Article or study type	Comparative observational studies Randomized controlled trials	Reviews Case reports Case series with less than 5 patients Commentaries Letters
Study population	Patients with gastrointestinal tumors in which fibroblast activation protein expression was measured	Other patients
Reported outcomes	Any of the following: Median overall survival 1-, 2-, 3-, or 5-year survival Histological differentiation (grading) Local tumor invasion Lymph node metastases Distant metastases	None of the outcomes mentioned as inclusion criteria
Language	English	Other languages

### Data Collection

Data from the included studies will be extracted separately by 2 authors and stored in a dedicated database. The following descriptive data will be documented for each selected study: first author, year of publication, inclusion period of the study, country and city where the study was conducted, sample size, and mean or median follow-up time. The distribution of the following patient characteristics will be documented: tumor type, histopathological tumor stage (using the UICC [Union for International Cancer Control] TNMG [tumor, node, metastasis, grade] classification system), presence and type of neoadjuvant therapy, presence and type of adjuvant therapy, FAP detection method, FAP antibody, FAP location, number of FAP-positive cases, and cutoff for overexpression.

The following predefined outcomes will be extracted: postoperative survival (overall and median survival, 1-, 2-, 3-, and 5-year survival rates), histological differentiation (grading), local tumor invasion (as defined in the included studies), lymph node metastases, and distant metastases. Subgroup analysis will be performed for location of FAP expression (tumor stroma or tumor cells or both) and tumor type (colon cancer, esophageal cancer, gastric cancer, hepatocellular carcinoma, cholangiocellular carcinoma, pancreatic cancer, and rectal cancer).

For each observational study, the risk of bias will be assessed using the ROBINS-I (Risk of Bias in Non-randomized Studies of Interventions) tool suggested by the Cochrane Collaboration [20]. For randomized controlled trials, the RoB 2 (Risk of Bias 2), the Cochrane risk-of-bias tool for randomized trials, will be used [21].

### Statistical Analysis

A meta-analysis comparing patients with and without FAP overexpression with the following outcomes will be performed: postoperative survival (overall and median survival, 1-, 2-, 3-, and 5-year survival rates), histological differentiation (grading), local tumor invasion (as defined in the included studies), lymph node metastases, and distant metastases. Random-effects models will be used. The ReviewManager (RevMan) software (version 5.4; Cochrane Collaboration) will be used. The magnitude of the effect estimate will be visualized by forest plots. Odds ratios will be calculated for binary data, and weighted mean differences and relative SD differences will be determined for continuous data. The 95% CI, heterogeneity, and statistical significance will be reported for each outcome. The chi-square and Kruskal-Wallis tests will be used to evaluate statistical significance. A *P* value of <.05 will be considered statistically significant. If studies do not report mean and SD, these will be calculated using the methods described by Cochrane Collaboration guidelines [22] and Hozo et al [23].

Sensitivity analyses will be conducted according to the ascertained risk of bias as described above. For these, all studies with a high or serious risk of bias will be excluded, and the analyses of the outcomes, as described above, will be conducted.

## Results

Database searches will commence in April 2023. Extraction of data from individual studies will be performed by May 2023. Data appraisal, preparation, summarization, and analysis will start in June 2023. The meta-analysis will be completed by December 2023.

## Discussion

This systematic review and meta-analysis will synthesize all available evidence on the clinical implications of FAP overexpression in GI tumors.

In recent years, several studies on this topic have been published. The last published meta-analysis on this topic dates to 2015 and included 15 studies about various solid tumors and only 8 studies focusing exclusively on GI tumors [24].

The main limitation of the present meta-analysis is that we do not expect randomized controlled trials on this topic to be available. Nevertheless, to ensure transparency and reduce the risk of bias, this review will be conducted according to the defined protocol presented here and will be reported following the recommendations stipulated in the PRISMA 2020 statement [18].

The expected results will provide new information on the prognostic value of FAP in GI tumors and thereby support health care professionals and patients in their decision-making, as well as aid in patient selection for multimodal cancer therapy.

## Abbreviations

α-SMA: α–smooth muscle actin
CAF: cancer-associated fibroblast
FAP: fibroblast activation protein
GI: gastrointestinal
PRISMA: Preferred Reporting Items for Systematic Reviews and Meta-Analyses
PROSPERO: International Prospective Register of Systematic Reviews
RoB 2: Risk of Bias 2
ROBIN-I: Risk of Bias in Non-randomized Studies of Interventions
TNMG: tumor, node, metastasis, grade
UICC: Union for International Cancer Control

## Data Availability

The data sets generated during this study will be available from the corresponding author upon request.
